# IQ Score of Children with Persistent or Perennial Allergic Rhinitis: A Comparison with Healthy Children

**Published:** 2014

**Authors:** Javad GHAFFARI, Ali ABBASKHANIAN, Masumeh JALILI, Jamshid YAZDANI CHARATI

**Affiliations:** 1 Allergy & Clinical Immunology Department, Mazandaran University of Medical Sciences, Sari, Iran.; 2 Department of Pediatric Neurolog, Mazandaran University of Medical Sciences, Sari, Iran.; 3 Masters in Psychology, Mazandaran University of Medical Sciences, Sari, Iran.; 4 Department of Biostatistics, Faculty of Health, Mazandaran University of Medical Science, Sari, Iran

**Keywords:** Intelligence Quotient, Children, Allergic Rhinitis, Prevalence

## Abstract

**Objective:**

Prevalence of allergies is different around the world. Allergic rhinitis is a common chronic disease in children.

Intelligence quotient (IQ) is an indicator of efficacy and many factors including chronic diseases may affect it. This study compares the IQs of children diagnosed with persistent or perennial allergic rhinitis with healthy children.

**Material & Methods:**

This was a comparative study that was conducted from June 2011–May 2013 in an academic referral clinic. In this study, 90 patients aged 6- to 14-yearsold who were diagnosed with persistent or perennial allergic rhinitis and were compared to 90 age and gender match healthy patients from their respective families. The Wechsler Intelligence Scale for Children was used to divide and calculate overall IQ, verbal IQ, and practical IQ. The t-test and chi square were used to analyze quantitative variables and qualitative variables, respectively.

**Results:**

In this study, out of total 180 children, 90 (50%) in the case group and 90 children (50%), the control group participated for IQ comparison. One hundred (57%) were male and 80 (43%) were female. The overall IQ for allergic rhinitis patients and healthy patients was 109.2 and 107.5, respectively. This difference was not considered significant. Furthermore, there was no significant difference between the IQ scores of males and females.

**Conclusion:**

Although allergic rhinitis is a chronic disease and effects quality of life, there were no identifiable negative effects on IQ.

## Introduction

The prevalence of allergies is different around the world. Allergic rhinitis (AR) or hay fever is a common nasal, eye, and upper respiratory membrane inflammatory disease that affects all ages. Prevalence of AR is 10–40% in different regions of the world as well as Iran (1, 2). According to the published studies, the prevalence of allergic diseases, such as AR, is increasing worldwide, especially in domestic regions. Over the past 20 years, the prevalence of AR in children has doubled. Etiologies of allergic disorders are multifactorial (genetic and environmental factors). Of environmental factors, aeroallergens play an important role in the exacerbation of allergic disorders especially in asthma and allergic rhinitis (3). Classical clinical manifestations of allergic rhinitis are sneezing, pruritus, congestion, and rhino rhea. AR is categorized according to its severity and duration into mild-intermittent, mild-persistent, moderate-to-severe-intermittent, and persistent. In persistent AR, the symptoms of patients last for more than 4-days in one week and longer than 4-weeks. 

Patients have classical symptoms such as sneezing, rhinorrhea, nasal congestion, and pruritus associated with alteration in quality of life such as learning, cognition, social behavior, and academic performance. In previous classifications, AR was divided into seasonal, perennial, and mixed. Allergic diseases have negative financial and social impacts on family and society. Although AR is not life threatening, it could be significant for morbidity by its effect on quality of life. The definition of intelligence is different among psychologists (4). These definitions include: mental capability of abstract thinking and reasoning; learning ability; accumulating knowledge; and the ability to solve problems. Chronic disease may affect cognitive abilities (3). The effects of chronic diseases such as epilepsy (5), cancer (**46**), and asthma (7) on the cognitive performance of children has been previously reported. COPD is a chronic lung disease that is a major risk factor for cognitive impairment due to chronic hypoxemia (8, 9). AR disease is a chronic disorder that may affect cognitive performance as well.

Of course, the severity and duration of the illnesses are important factors. ARs association or effect on the cognitive performance of children remains unclear.

Marshal indicated only atopic subjects exhibited declines in verbal learning, slower decision-making, and psychomotor speed on both simple and choice reaction time tests and lower positive affect during their allergy seasons in comparison to other seasons. Atopic subjects did not demonstrate declines in the ability to sustain attention. Biochemical mechanisms may have caused these changes (10).

However, few studies have examined the relationship between IQ and AR. By defining this relationship and controlling AR, effective measures can be done to improve the quality of life and academic performance of these children.

Therefore, we assess the IQ of children aged 6 to 14 who were diagnosed with persistent AR and compared them with a similar healthy group of children. 

## Material & Methods

This is a comparative study that was conducted from June 2011–March 2013 in the Tuba clinic and Boali Sina Hospital in Sari, Mazandaran University of Medical Sciences, Iran. Ninety children with AR participated in this study. The children were 6- to 14-years old. The control group consisted of 90 healthy children. The data collected included age, gender, previous history of asthma, and medical history of patients from interviews with parents and from the medical histories of patients. 

All patients under went skin prick testing for aeroallergen extracts. We chose patients who had at least one positive test. The Wechsler intelligence scale for children was used to calculate IQs. The inclusion criteria of patients included: 1. age between 6 to 14 year old, confirmation of AR from clinical evaluation, a positive skin prick test, history of persistent AR at least for 2-years; and 2. No history of other chronic diseases such as heart, brain, kidney, and liver diseases. Consecutive sampling was used. All patients and control groups were selected from within their family.

Exclusion criteria included: children younger than 6-years of age or older than 14-years of age, previous history of acute or chronic diseases other than AR, neurological disorders, and known mental retardation. The control group was chosen from healthy children among the patients’ families. They had no current or previous history of allergies and no allergy related diseases. The IQ test for both groups was performed by the same person in a similar situation. Before treatment, all patients underwent IQ testing. The study was approved by ethics committee of Mazandaran University of Medical Sciences and informed consent was taken from all patient`s parents.

**Research tools**

The Wechsler intelligence scale for children is one the most accepted tests and is widely used to evaluate the IQ of children. The Wechsler Intelligence Scale for Children (WISC), developed by David Wechsler, is an individually administered intelligence test for children between the ages of 6 and 16 and provided scores for Verbal IQ (VIQ), Performance IQ (PIQ), and Full Scale IQ (FSIQ)(11,12). This test was used by a split half method and its reliability was /97 for overall IQ, /97 for verbal IQ, and /93 for practical IQ (11). The validity and reliability of this test was approved by Shiraz University (12). This test was performed by an MA and a Ph.D. psychologist. A total of 212 people have been tested, but seven patients were excluded due to failure to follow instructions. All tests were performed at the Tooba Clinic Center, Mazandaran University of Medical Sciences, Sari, Iran.

**Data analysis**

SPSS 15 was used to analyze the data. The t-test was used to compare the calculated scores of the groups. 

Qualitative variables were analyzed by the chi square test. The f-test was used for quality of variance of groups for applying the T-test. A p-value less than 0.05 were considered significant.

## Results

The study population included 180 children. The AR group and the healthy group consisted of 90 (50%) and 90 (50%), respectively, with a mean age 8.5 ± 2.32 and 8.89 ± 2.65 years old, respectively (p=0.134). In both groups 100 (57%) were male and 80 (43%) were female. 

The mean age of the participants was 8.75 ± 2.45 year old (Table1).

Table 2 Shows The Mean IQ and The Statistical Indicators of IQ Scores of Each Group.

Table 3 shows the t-test was used to compare the mean IQ of the two groups. The difference was not significant. 

The relationships of these groups with variables such as gender were evaluated by chi square test and Table 4 shows the result.

**Table1:** Shows the Gender Distribution in Allergic Rhinitis and Healthy Groups

**Sex **	**Male **	**Female **	**Total**
**Cases**	58(64%)	32(36%)	90(50%)
**Control**	42(47%)	48(53%)	90(50%)
**Total**	100(57%)	80(43%)	180(100%)

**Table 2:** Shows the Mean IQ Aand the Statistical indicators of IQ Scores of Each Group

	**Number**	**mean**	**Std. deviation**
Verbal:			
Cases	90	107. 27	12.368
Control	90	104.68	11.175
Practical:			
Cases	90	110.23	13.767
Control	90	108.60	11.737
Both (verbal and practical):			
Cases	90	110.02	12.968
Control	90	107.58	10.895

## Discussion

According to our research from different sources, the numbers of related articles to the IQ scores of children diagnosed with AR are limited (13). Previous studies have shown that chronic illnesses such as epilepsy (5) tend to adversely affect the cognition in children whereas extrinsic asthma does not adversely affect cognition in children (14, 15).

As shown in our study, the mean overall IQ score of AR patients was statistically similar to healthy children (p=0.106). In addition, there was no significant difference in the verbal IQ (p =0.348) and practical IQ (p=0.248) between the two groups. Furthermore, the mean IQ score of patients and healthy children was not statistically significant.

In a study conducted in Nigeria, 34 patients had atrophy were compared to 94 healthy participants and no the differences in IQ scores were observed between gender and the two groups. The mean IQ score of younger participants was lower than for older participants (16). 

However, in this study, after eliminating gender, age, and study level of the patients, the mean IQ score of patients was lower than the control group. However, our study and other studies have shown that asthma and allergic rhinitis does not alter cognitive function (13, 16). Keremer et al conducted a study on 26 AR patients and compared them with 36 healthy people aged from 16 to 65 years (adult study). They showed that although psychological symptoms such as somatic disorders, anxiety, sleep disorders, and depression was higher among patients, their cognitive function was similar to the healthy group (17). This study also showed that the cognitive function of patients with AR was not significantly different from healthy individuals.

**Table 3:** T-test was used to compare the mean IQ of the two groups. The difference wasn’t significant

	**Levene`s test (F)**	**p-value**
Verbal:		
Equal variances assumed and not assume	0.884	0.348
Practical:		
Equal variances assumed and not assumed	1.346	0.248
Both (verbal and practical):		
Equal variances assumed and not assumed	2.641	0.106

**Table 4:** IQ Score Distribution Based on Gender between Two Groups

	**Verbal**		**Practical **		**Both**	
	**mean**	**Ste.Deviation**	**mean**	**Ste.Deviation**	**mean**	**Ste.Deviation**
**Male:**						
**Cases(58) **	106.48	12.552	107.93	14.037	108.26	13.035
**Control(42) **	102.95	11.849	107.33	12.993	106.00	12.384
**Female:**						
**Cases(32) **	108.69	12.092	114.41	12.407	113.22	12.412
**Control(48) **	106.19	10.441	109.71	10.531	108.96	9.317

**Table 5:** IQ score verbal, practical and overall based on gender

**Gender**	**IQ Score Equal Variances**	**Levene`s test**	**(F) p-value**
**Male:**	Verbal: Equal variances assumed and not assumed	0.368	0.545
	Practical: Equal variances assumed and not assumed	0.123	0.727
	Both (verbal and practical):Equal variances assumed and not assumed	0.230	0.633
**Female:**	Verbal:Equal variances assumed and not assumed	0.714	0.401
	Practical: Equal variances assumed and not assumed	1.499	0.225
	Both (verbal and practical): Equal variances assumed and not assumed	3.037	0.085

On the other hand, Marshal and colleagues showed that data transmission speed of patients involved with allergies was significantly slower than the healthy group. They stated that this result was only seen when the Hick test was performed. Other tests showed no significant differences. This study showed that the speed of cognitive procedures was significantly lower among patients with a ragweed allergy. However, their attention and recent memory was similar to the healthy group. 

Some patients had problems in their working memory. 

In the end, they concluded that allergic reactions caused significant problems in some patients (13).

Similar studies should be performed with a larger sample size and among patients with severe asthma who have a higher probability of developing hypoxia.

**In conclusion, **this study showed that children with persistent AR exhibit an IQ similar to healthy children.

Limitations of this study are only moderate/severe allergic rhinitis and with patients who are between 6–14 years of age. Therefore, we suggest further studies with different severities of AR and a wider range of ages. 

Our patients were from urban and rural communities that might have some effect on the IQ scores in patients with AR.

## Conflict of interest

The authors reported no conflict of interests.

## Author’s contribution

Dr Ghaffari: Approved proposal, selected patients, and writting the paper

Dr Abbaskhanian: Approved proposal, conducted IQ tests, and wrote paper

Dr Jalili: Conducted IQ tests

Dr Yazdani Charati: Conducted data analysis
